# Immune priming: the secret weapon of the insect world

**DOI:** 10.1080/21505594.2020.1731137

**Published:** 2020-02-21

**Authors:** Gerard Sheehan, Gemma Farrell, Kevin Kavanagh

**Affiliations:** Department of Biology, Maynooth University, Maynooth, Ireland

**Keywords:** *Galleria* larvae, *in vivo* model, infection, immunity, priming

## Abstract

Insects are a highly successful group of animals that inhabit almost every habitat and environment on Earth. Part of their success is due to a rapid and highly effective immune response that identifies, inactivates, and eliminates pathogens. Insects possess an immune system that shows many similarities to the innate immune system of vertebrates, but they do not possess an equivalent system to the antibody-mediated adaptive immune response of vertebrates. However, some insect do display a process known as immune priming in which prior exposure to a sublethal dose of a pathogen, or pathogen-derived material, leads to an elevation in the immune response rendering the insect resistant to a subsequent lethal infection a short time later. This process is mediated by an increase in the density of circulating hemocytes and increased production of antimicrobial peptides. Immune priming is an important survival strategy for certain insects while other insects that do not show this response may have colony-level behaviors that may serve to limit the success of pathogens. Insects are now widely used as *in vivo* models for studying microbial pathogens of humans and for assessing the *in vivo* efficacy of antimicrobial agents. Knowledge of the process of immune priming in insects is essential in these applications as it may operate and augment the perceived *in vivo* antimicrobial activity of novel compounds.

**Abbreviations:** 1,3-dibenzyl-4,5-diphenyl-imidazol-2-ylidene silver(I) acetate; SBC3: antimicrobial peptides; AMPs: dorsal-related immunity factor; DIF: Down syndrome cell adhesion molecule; Dscam: Lipopolysaccharide; LPS: Pathogen-associated molecular patterns; PAMPS: Patterns recognition receptors; PRR: Prophenoloxidase; PO: Toll-like receptors; TLRs: Toll/IL-1R; TIR, Transgenerational Immune Priming; TgIP: Tumor necrosis factor-α; TNF-α.

## Introduction

Insects are an extremely successful and diverse group of animals that inhabit almost all habitats and ecosystems on Earth [1,2]. Insects play essential roles in the pollination of flowering plants, production of silk and, in certain societies, can be a food source. Insects are also a significant cause of crop losses and food wastage, and are important vectors of infectious diseases (e.g. malaria, dengue fever, Zika) which account for high levels of morbidity and mortality particularly in the developing world [3]. The success of insects has been attributed in the past to their rapid rates of reproduction, their relatively short lifespan and their ability to rapidly adapt to changing environments [4]; however, recent research has highlighted the possession of a highly effective immune response in protecting them from pathogens [5]. The insect immune system consists of interconnected cellular and humoral responses that quickly identify and destroy or immobilize invading pathogens [6,7]. The cellular immune response is mediated by hemocytes which can phagocytose and kill pathogens or entrap large pathogens (e.g. eggs of parasitic wasps) and immobilize them [8]. A wide range of hemocytes (e.g. plasmatocytes, granulocytes, coagulocytes, spherulocytes, oenocytoids) [9,10] exist in insects but a full definition of the different subclasses is not yet established. Pattern-recognition receptors (PRR) recognize pathogen-associated molecular patterns (PAMPS). Many PRRs exist in insects such as LPS binding proteins, β-1,3 glucan binding protein, and peptidoglycan recognition protein [11,12]. Two distinct members of the NF-κβ family of inducible trans-activators, DIF (dorsal-related immunity factor) and Relish are responsible for the regulation of antimicrobial peptide production [13–15].

The humoral immune response of insects involves the production of antimicrobial peptides by hemocytes and cells of the fat body and the production of melanin. Insects produce a wide range of antimicrobial peptides (AMPs) and these can display potent antibacterial and/or antifungal activity. Insect AMPs include lysozymes which are cationic proteins that target and kill Gram-positive bacteria through hydrolyzing the peptidoglycan β-(1,4) glycosidic bonds in the bacterial cell wall [16] and defensins which are characterized by a group of cysteine-rich cationic peptides that contain several disulfide bridges and cecropins which are amphipathic α-helical AMPs of 11 amino acids in length that have the ability to target and kill bacteria and filamentous fungi [17,18]. Cecropin has antibacterial activity against multidrug-resistant *Acinetobacter baumannii* and *Pseudomonas aeruginosa* induces apoptosis of *Candida albicans* cells and possess immunomodulatory effects on macrophages [19,20].

The immune system of insects demonstrates many similarities to the innate immune response of mammals. Insect hemocytes show structural and functional similarities to mammalian phagocytic cells (e.g. neutrophils, macrophages) in that both can phagocytose, produce superoxide and degranulate [17]. The mammalian complement cascade also possesses several similarities to melanization in insects [18,19]. Melanization in insects involves a number of cascades that must be carefully regulated due to the production of toxic and reactive intermediates which may be detrimental to the host. Excluding the final step in which melanin is produced, the prophenoloxidase (proPO) system displays similarities to the complement system of vertebrates. In both the complement system of mammals and the proPO system of insects, there is production of cytotoxic and opsonic components [20]. Furthermore, there is some similarity between the sequences of insect proPO and the mammalian complement proteins C3 and C4 [21].

**Figure 1.**
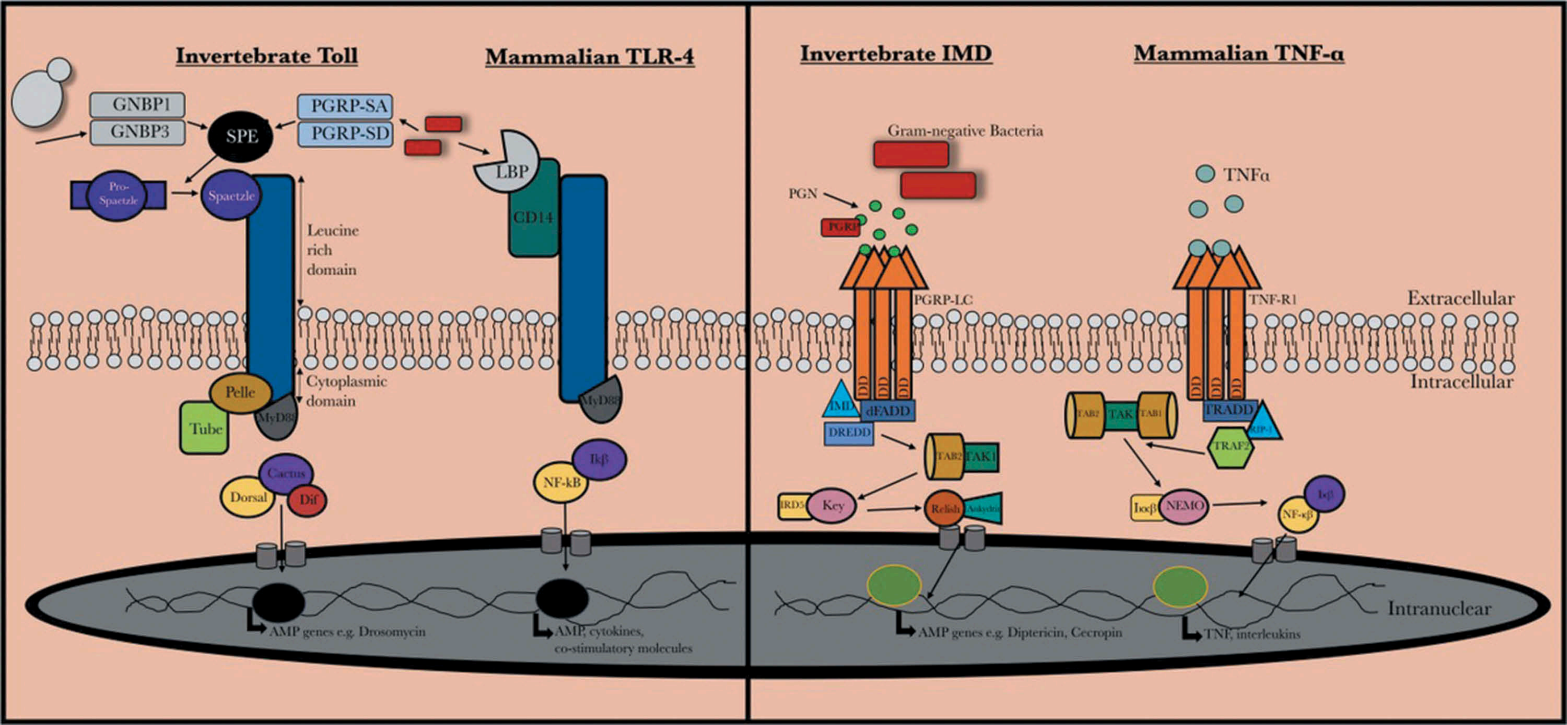
Comparision between mammalian and invertebrate Toll/Toll-like receptor and IMD/TNF-α signaling. Upon activation of invertebrate Toll receptor and the homologous Toll-like receptor in vertebrates, a cascade is induced where the homologous transcription factors Nf-κB and Dif are activated in vertebrates and invertebrates, respectively. The IMD pathway is activated by binding of peptidoglycan (PGN) to peptidoglycan-recognition proteins (PGRPs) which results in recruitment and formation of an IMD, dFADD and DREDD complex and results in IMD cleavage and activation of TAB2/TAK1. This results in Relish phosphorylation and ultimately the production of AMPs (e.g. cecropin). In mammals, TNF-α is bound by the tumor necrosis factor receptor 1 (TNF-R1) which results in recruitment of RIPP, FADD, and caspase 8. NF-κB is released from its inhibitor protein (IκB) via phosphorylation by IKK complex which results in NF-κB translocation to the nucleus resulting in pro-inflammatory cytokine production.

Pathogen recognition in insects and mammals can be achieved by a range of bacterial lipopolysaccharide, peptidoglycan and fungal β-1,3 glucan recognition proteins [22,23] and insects and mammals produce similar antimicrobial proteins (e.g. defensin, lysozyme). Toll-like receptors (TLRs) are a group of type I transmembrane receptors that play a role in innate humoral immunity in both insects and mammals. Homologies between these receptors can be observed between the cytoplasmic Toll/IL-1R (TIR) domain of both mammalian Toll-like receptors and the *Drosophilia* T receptor. There are also significant similarities between the insect immune deficiency (IMD) pathway which recognizes components of the bacterial cell wall such as peptidoglycan, resulting in the activation of a cascade that ultimately produces AMPs and the mammalian tumor necrosis factor-α (TNF-α) pathway (Figure 1). Both the TNF-α and IMD pathways ultimately result in the production of the homologous transcription factors NF-κB and Relish, respectively [24,25].

Due to these conserved features of the immune responses many insects (e.g. *Galleria mellonella, Drosophila melanogaster, Manduca sexta, Bombyx mori*) have been widely employed to assess the virulence of fungal and bacterial pathogens and the results of studies correlate with those obtained using mammals [26,27]. *D. melanogaster* is a well-established model due to the range of molecular tools available such as genome editing and mutant library availability and the results obtained using flies have been translated into modern medicine, e.g. discovery of Toll in *Drosophila* resulted in the identification of toll-like receptors (TLR) in mice and humans [28,29]. Insects have also been used to measure the *in vivo* efficacy of antimicrobial agents [30,31] and to evaluate the *in vivo* toxicity of chemicals [32–35].

Insect do not possess a system equivalent to the antibody-mediated adaptive immune response of vertebrates however some insects display a phenomenon called “immune priming” in which prior exposure to a non-lethal inoculum of a pathogen, pathogen-derived material or stress event stimulates the immune response to render the insect resistant to a normally lethal infection a short time later [36]. While immune priming is not equivalent to the adaptive immune response of vertebrates, it does offer a degree of protection to subsequent infections but is metabolically costly to initiate and maintain [37]. The protection afforded by immune priming may protect insects from infection, but this process is also important to be aware of when using insects as *in vivo* models.

## Immune priming in different insect species

Immune priming in insects has the advantage of giving protection from a subsequent potentially lethal infection but is costly to maintain and can result in death in the absence of feeding [38]. Immune priming can be recognized by an increase in the density of circulating hemocytes and the elevated abundance of AMPs in the insect hemolymph [39]. The increased hemocyte density arises from the activation of sessile hemocytes which are normally attached to the inner surface of the cuticle rather than *de novo* synthesis and an increased density of circulating hemocytes has been correlated with protection from infection [40–43].

Pre-exposure of *G. mellonella* larvae to the yeast *C. albicans* protects against a subsequent infection by this yeast that would normally prove fatal. Interestingly, administration of *Saccharomyces cerevisiae* to larvae can also induce the same protective effect against *C. albicans* infection thus indicating that the response may be a generalized antimicrobial response. Immune priming in this case was correlated with an increase in the expression of genes for a range of antimicrobial peptides with strong antifungal activity (e.g. *gallerimycin, galiomicin*) [41]. Inoculation of *D. melanogaster* with a sub-lethal dose of *Streptococcus pneumoniae* protected against a subsequent lethal inoculum and this was mediated by the enhanced phagocytic ability of hemocytes [44]. In *Bombus terrestris,* the protection and specificity of immune priming can last up to 22 d, which is long after transcription and the elevated production of antimicrobial peptides has ended [45].

Components of microbial cell wall can also induce immune priming in *G. mellonella* larvae. Laminarin [41], and β-glucan administration can induce protection against subsequent infection by *C. albicans* [46]. In the case of glucan-induced immune priming, the degree of the protection was correlated with the dose of the glucan administration with low inoculum inducing 60% survival at 24 h while a high dose of glucan resulted in 100% survival of larvae. There was a strong correlation between dose of glucan and extent of the increase in the hemocyte density and antimicrobial peptide abundance [46].

While immune priming has obvious survival advantages for insects it is not found in all insect species. Immune priming in *Formica selysi* (ant) workers following challenge with the fungal pathogen *Beauveria bassiana* was short term [47] raising the possibility that colony living removes the necessity of having a prolonged immune priming response as other compensatory mechanisms may be operating. Ants have an abundance of collective defenses that limit the spread of a pathogen through colonies [48] and as such, the individual cost of investment in these collective defenses may outweigh the cost required for an individual priming event. Honeybees (*Apis meliferra*) show less individual investment in a primed immune response but rather, colonies experience elevated temperatures as part of a colony-level response in order to prevent infection against a common heat-sensitive pathogen, *Ascosphaera apis* [49].

Immune priming is metabolically costly to induce and maintain consequently certain insects may not need it if immune protection is achieved by life within a colony where colony-level immune protection is active. A reduction in the expression of arylphorin and lipoprotein was demonstrated in *Bombyx mori* when a calorie restriction was applied to the insect’s diet [50]. In contrast, *Tribolium casteneum* deprived of food demonstrated increased expression of selected stressor specific microRNAs in a gender-specific manner thus suggesting that different types of insects react differently to nutrient deprivation [51]. Dietary restriction can lead to an altered expression of a number of immune-related genes and a delayed up-regulation of antimicrobial genes in *D. melanogaster* [52]. While immunological priming has clear benefits to an insect and may allow it to withstand a subsequent lethal infection it does carry a biological cost and, in the absence of compensatory feeding, can be fatal [38].

## Immune priming as a result of exposure to anti-microbial agents

Introduction of foreign material into the hemocoel of *G. mellonella* larvae can trigger an increased immune response and resistance to subsequent infection. Enhanced larval survival was seen in *G. mellonella* larvae administered a dose of caspofungin before an inoculation with *C. albicans*. The increased resistance was mediated by an elevated hemocyte density and expression of genes coding for *transferrin* and *IMPI* and by the increased abundance of apolipophorin and prophenoloxidase [30]. Interestingly, administration of caspofungin also resulted in the larvae showing increased resistance to *Staphylococcus aureus* infection even though caspofungin has no inherent antibacterial activity. This indicated that the priming event may be a generalized immune response producing resistance to fungal and bacterial infection.

## Thermal and physical stress as immune priming agents

**Figure 2.**
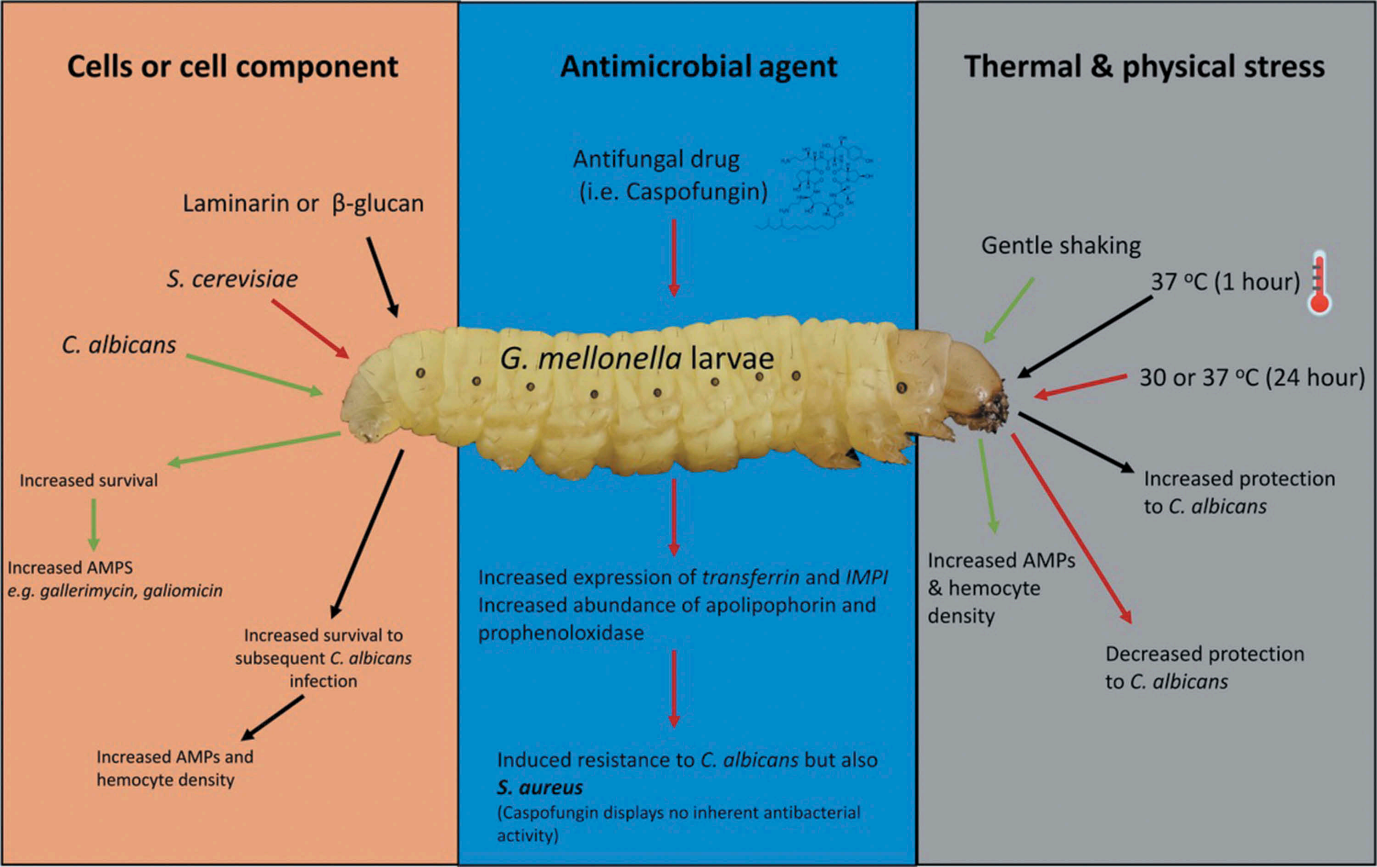
Summary of immune priming in *G. mellonella* larvae. The effect of cells or cell component, antimicrobial agents or thermal and physical stress on immune priming in *G. mellonella* larvae. Components of the fungal cell wall (laminarin/β-glucan) or a sub-lethal *C. albicans* or *S. cerevisiae* infection protect from a subsequent potentially lethal infection by an increased abundance of AMPs and the number of circulating hemocytes. The antifungal agent caspofungin induced increased resistance to *S. aureus* infection. Gentle shaking and a 1 h 37°C incubation induces immune priming in larvae.

Physical factors such as incubation temperature or agitation can also induce immune priming. Gentle shaking *G. mellonella* larvae in cupped-hands for a short period of time can induce an increase in the expression of AMPs and in the density of circulating hemocytes and may be mediated by disruption of the integrity of the gut epithelium allowing bacteria enter the hemocoel [6]. Alterations in incubation temperature can induce immune priming. Incubation of larvae at 37 ^o^C for 1 h leads to increased protection against subsequent challenge with *C. albicans*. In these instances, immune priming offers short-term protection to larvae. Browne et al. [53] found that when the larvae of *G. mellonella* were incubated at 30°C or 37°C for 72 h, they showed decreased survival compared to larvae exposed to the same conditions for a shorter period of time (24 h) prior to infection [53]. The density of hemocyte populations and the level of AMPs reached a peak at 24 h and then declined as the cost and maintenance of the primed response may outweigh its benefits. The reduced levels of both hemocytes and AMPs correlated with the reduced survival rates of the inoculated larvae. Immune priming in *G. mellonella* larvae by components of the microbial cell, anti-microbial agents and physical and thermal stress is summarized in Figure 2.

## Specificity in the primed response

Insect lack an antibody-mediated adaptive immune response as occurs in vertebrates however immune priming does show some functional similarities to aspects of the vertebrate adaptive immune response [54]. The primed response may be protective in a species-specific manner in scenarios in which there exists a specific fungal/bacterial pathogen that poses a high-risk/repeated risk of re-infection toward the insect host [55].

Sadd and Schmid-Hempel [45] and Lemaitre et al. [56] highlighted the ability of insects to mount a response that is specific to the particular pathogen. However, in order to respond specifically to that pathogen, the host must possess a system which allows the rapid identification and discrimination of pathogens [45,56]. While the exact mechanism(s) of recognition have not yet been fully elucidated, once a species has evolved means of recognition toward a particular antigen, there is likely to be a selection for memory. Having a specific memory of a pathogen would be advantageous to the insect in scenarios where it may be exposed to the same pathogen multiple times throughout its life [54]. The memory of past infection would facilitate a rapid immune response, and this would subsequently promote clearance of infection, ultimately contributing to the enhanced survival of the insect. However, there is little evidence to suggest that there is an element of memory in the insect immune system [57].

The insect immune system is capable of responding in a species-specific manner to a pathogen if that particular pathogen is encountered on more than one occasion. True specificity in immune priming should occur when there is a heterologous challenge between two homologous challenges and that the initiation of a primed response toward the second homologous infection is induced by a “memory” of the initial infection and not due to a sustained immune response from the heterologous or intervening challenge [58]. Lemaitre et al. [56] characterized *Drosophila* host defenses, where the specific expression of different antimicrobial peptide genes following inoculation by a variety of pathogenic microorganisms was studied. It was found that *Drosophila* which are naturally infected by entomopathogenic fungi generate an adaptive response in which there was a specific induction of AMPs with a specific antifungal role. It is believed that this response was mediated specifically via the activation of the Toll pathway (Figure 1). This Toll pathway controls expression of the *drosomycin* antifungal gene in *Drosophila*. Antibacterial peptides are induced via a pathway that involves the immune-deficiency gene or as a cumulative result following the activation of both IMD and Toll pathways (Figure 1) [56]. However, in the case of fungal infections in *Drosophila* they are capable of mounting an adaptive response that is specific to the particular fungal infection and thus may be classified as a specific priming response. While the above study examined the generation of a specific priming response toward fungal pathogens, similar observations were also recorded for bacterial pathogens, but it does not appear to be the case for all invertebrate infections. In some studies, the AMP profile in response to infection was nonspecific to the presented challenge [59]. The gene responses in *D. melanogaster* have been demonstrated to be altered in response to invasion of a specific pathogen [60,61]. It has also been shown that insects may be primed against infection by certain pathogens based on prior exposure [41,62]. Also, in *D. melanogaster*, infection with *Streptococcus pneumoniae* did not prime the humoral response but activated plasmatocyte phagocytosis and this was identified as a key effector against a secondary response to the same pathogen in primed flies [44].

*Tenebrio molitor* was afforded a high level of protection against several Gram-positive and Gram-negative bacterial infections when initially primed with Gram-positive bacterial species as a result of a persistent antimicrobial response. When the experiment was repeated, substituting a Gram-negative bacterium as the priming agent, a primed response was observed but ultimately, the primed response induced by Gram-positive bacteria yielded a higher survival rate in the insect [63]. *T. molitor* mothers that were challenged with either Gram-negative or Gram-positive bacteria produced eggs which demonstrated enhanced antimicrobial activity against Gram-positive bacteria regardless of the nature of the priming species, due to the transfer of tenecin-1 from the mother to the offspring via the egg. These results suggest that Gram-positive pathogens may have acted as a very important driving force for the selective evolution of *T. molitor* priming, as these major entomopathogenic pathogens persist successfully in the external environment around *T. molitor* and thus there is a higher possibility of infection [64]. The above example highlights the presence of a selective pressure on the evolution of the primed response in *T. molitor* to act in a specific manner upon encountering a Gram-positive bacterium which poses a significant risk of re-infection to the host. However, the response itself acts in a relatively nonspecific manner in which the insect is afforded protection from a range of pathogens (Gram-positive and Gram-negative) for a short-term heightened defense but this is achieved only when the specific Gram-positive bacterial pathogen is first encountered.

*Bombus terrestris* has also demonstrated some specificity in the primed response. Bumblebees were initially primed by exposure to Gram-negative bacteria or by one of two closely related Gram-positive bacteria. Subsequent challenging of the bees with a homologous bacterial species or by any one of the other two bacterial species to which they were not previously exposed showed that the bees presented significantly higher levels of survival when faced with a homologous secondary infection as opposed to a heterologous secondary infection even in the case where the heterologous infection involved two closely related bacterial species [45].

Immune stimulation of honeybees, *Apis mellifera*, by inoculation with lipopolysaccharide (LPS) alters the expression of genes coding for *defensin* and alters the behavior of challenged bees [65]. A super-spreading hemocyte has been recorded after microbial challenge of *Manduca sexta* but not following wounding [66] indicating a differential response to a microbial challenge compared to a physical insult.

When exposed to a microbial pathogen the expression of a wide range of AMPs may be required whereas the response to a physical challenge (e.g. shaking, temperature variation, wounding) may only require the induction of a subset of antimicrobial peptides. Presumably, a microbial infection is a greater threat in that the microbe may proliferate and disseminate within the insect while a mild physical stress (e.g. damage to the cuticle, shaking) may be self-limiting, unless there is the entry of a pathogen, and may not pose an immediate threat to the insect’s survival. The administration of fungal cell wall material to *G. mellonella* larvae resulted in the activation of a number of genes [41] however only a subset of genes was activated when insects were physically challenged although both treatments lead to immune priming [46].

Administration of a low dose of *A. fumigatus* conidia to *G. mellonella* larvae leads to activation of the cellular immune response but an inoculum of 1 × 10^5^ conidia lead to the increased expression and binding of immune-related proteins, which are components of the humoral immune response, as well as increased hemocyte density [39]. It is possible that a low-level infection can be eradicated by increasing the hemocyte density but that larger inocula may require elevated hemocyte densities as well as increased abundance of AMPs. This indicates that the insect immune system may be capable of sensing the extent of microbial challenge and mounting a “proportionate” response in order to ensure survival but minimize the use of resources.

Research is actively focused on determining the recognition mechanisms which would facilitate specificity and targeted gene expression toward certain pathogens in the insect immune-primed response. This is expected to be related to the mechanisms of pathogen recognition by way of PRRs as seen in vertebrates [14,56]. A number of molecules have been identified as candidates for receptor diversity, facilitating the development of a specific adaptive response in the insect immune response. While certain molecules, such as Dscam are considered good candidates allowing for receptor diversity in the insect immune system, it is possible that diversity is limited when compared with the vertebrate adaptive response which has the ability to recognize an infinite number of potential antigens [67]. Assuming the limited potential diversity of pathogen receptors by insects, it is reasonable to assume that the presence of targeted specificity within the primed response of insects is primarily restricted to a particular range of pathogens. This indicates that not all pathogens that an insect encounters present the same possibility of re-infection or pose the same risk to insect success and thus a primed immune response, and in particular a pathogen-specific primed response, would be more cost-effective when directed at only pathogens which pose the highest threat of re-infection and death [55].

## Transgenerational immune priming in insects

A form of immunological memory in insects has been identified and is known as Transgenerational Immune Priming (TgIP). TgIP involves the passing of a protective effect from the parent insect to its offspring [68]. The exact mechanisms that regulate this effect have not yet been fully characterized but can involve fragments of bacterial cell wall material being laid with eggs [69]. The *Paenisbacillus* fungal infection is lethal to the honeybee, *Apis melliferia*, and experiments have shown that when queen bees were infected with heat-killed *P. larvae*, the offspring showed a 26% reduction in the level of larval mortality when compared to the progeny of control queens. In addition, the offspring of the immune primed honeybees contained a threefold increase in the level of differentiated hemocytes which are involved in the production of AMPs employed for bacterial clearance [70]. TgIP may be a mechanism of long-lasting protection, but there is an unresolved issue relating to the number of generations to which this memory-like effect persists. The Indian-meal moth, *Plodia interpunctella*, challenged with the *Plodia interpunctella* granulosis virus demonstrated inherited protection in the F_2_ but not in the F_3_ generation [71] while priming of *Tenebrio molitor* with LPS of *Escherichia coli* followed through for two generations [68].

The ability of the vertebrate adaptive immune system to retain the memory of past infection promotes the development of a biphasic response upon pathogen re-encounter. The only recorded investigation into the existence of the biphasic response in the invertebrate immune system was conducted by Contreras-Garduno *et al*. [72] using *Anopheles albimanus* [72]. This study found that following the priming of *A. albimanus* with *Plasmodium berghei*, a second exposure showed that the level of the gambicin, attacin and cecropin antimicrobial peptides were elevated compared to the initial exposure despite allowing sufficient time for hemocyte and AMPs to return to basal levels with pathogen clearance following the first exposure. In the eukaryotic cell, *hnt*, a zinc-finger protein, is a key component that facilitates Notch in the switching of the cell cycle from mitosis to the endocycle. There was a +8.1 fold increase of *hnt* in mosquitoes primed with live ookinetes. These elevated levels imply that the midgut cells of the primed mosquitoes have an increased number of gene copies facilitating the rapid production of effector transcripts and proteins, supporting a heightened readiness for infection which then allows for a rapid and effective response through this adaptive response [73].

Interestingly, TgIP can produce different results depending on the developmental phase of the insect. For example, in *M. sexta*, non-primed offspring develop and grow more quickly, and this indicates that priming probably evolved in insect species that were subjected to an environment containing pathogens. However, primed offspring that become adults (females) lay fewer eggs [74]

## Conclusion

Immune priming gives some insects the ability to withstand potentially lethal infections if previously exposed to a sub-lethal inoculum or a stress event. It is mediated by an increase in elements of the cellular and humoral immune responses but it is not found in all insect species. The increased use of insects (e.g. *G. mellonella, D. melanogaster*) as *in vivo* models for evaluating activity of antimicrobial drugs is welcome and has many advantages. However, researchers need to be aware that compounds with no inherent antimicrobial activity (e.g. glucan, LPS) can trigger immune priming and render an insect resistant to a pathogen. In these circumstances, some inactive compounds may be incorrectly classified as having *in vivo* antimicrobial activity. Even in the case where a compound does display *in vivo* activity, it may trigger immune priming which is a second line of defense in the insect. Thus, it is essential that the ability of novel antimicrobial compounds be assessed for their immune priming effects before true *in vivo* activity can be assigned to them.
